# Therapeutical Notes

**Published:** 1900-06-16

**Authors:** 


### THERAPEUTICAL NOTES.

### A Cardiac Tonic.

Carrieu recommends the following in valvular disease when arterio-sclerosis is also present: Spartein, grains 1½; iodide of potash, grains 8 to 15. This amount is to be made into a draught, and taken in the course of a day in divided portions. Digitalis, he considers, is undesirable when we wish to avoid increasing the blood pressure, but the iodide benefits the tension, while the spartein acts on the heart without affecting the vessels.—*La Semaine Médicale*, No. 45, 1899.

### Psoriasis.

The following is said to be recommended by Talamon (*New York Med. Jour.*, January 6th):—

℞. Salicylic acid, grains 45.
Ichthyol, grains 150.
Pyrogallic acid, grains 90.
Lanoline or vaseline, grains 1,500.
Mix. S. To be painted on the spots.

Dr. M. Hodara paints the affected parts every two or three days with the following solution, and dresses them with a 1 per cent. ichthyol ointment on the intervening days:—

Chloroform, 6 drachms.
Glycerine, 5 drachms.
Chrysarobin, 40 grains.
Ichthyol, 40 grains.
Salicylic acid, 40 grains.
Mix. To be used as a paint.

For the face and scalp he employs an ointment, containing chrysarobin (1 in 400), ichthyol (1 in 100). This is applied every night, and removed in the morning by rubbing with olive oil. For a few days while the treatment is being carried out the parts must not be washed with soap and water.—*Merck's Archives*, Dec.

### Formaldehyde in Phthisis.

J. Lardner Green advises the inhalation of a spray solution in early phthisis. Each application lasts 10 to 15 minutes, and may be repeated four times a day. He makes use of the following formula:—

Formaldehyde, ʒi.

Glycerin, ʒiv.

Aquæ destillat., ℥v.

M. Make a spray.

—*Lancet*, 1899, p. 521.

### Tapeworm.

Marr and Quanjer believe that castor oil should not be given with male fern, because the latter forms a poisonous and easily-absorbed compound with it. Quanjer gives early in the morning some aperient water, then five grains of filicin in divided doses, and, if necessary, another dose of the water. Marr administers an aperient the day before, and 20 or 35 grains of extract of male fern in the morning fasting, with wine and water. Both keep the patient in bed till the bowels have acted. Where male fern causes vomiting the black oxide of copper is given in pill form for a fortnight, and a final dose of castor oil completes the course. Sour foods should be avoided during the whole time. (*Med. Rec.*, 64, 1899.) P. Manson, in Allbutt's "System of Medicine," speaks highly of a decoction of pomegranate bark or of the pelleterine made from it for adults, and of pumpkin seeds for children. A drachm of chloroform well diluted and taken in divided doses has been found at times a valuable remedy; and recently it has been stated that if a part of the worm projects from the anus and a little morphia be injected into it, the whole animal is quickly killed and expelled.

